# Relationship between level of CPR training, self-reported skills, and actual manikin test performance—an observational study

**DOI:** 10.1186/s12245-018-0220-9

**Published:** 2019-01-10

**Authors:** Inger Lund-Kordahl, Maria Mathiassen, Jørgen Melau, Theresa Mariero Olasveengen, Kjetil Sunde, Knut Fredriksen

**Affiliations:** 1 Anaesthesia and Critical Care Research Group, Department of Clinical Medicine, UiT The Arctic University of Norway, N-9037 Tromsø, Norway; 2 Division of Pre-hospital Services, Vestfold Hospital Trust, N 3103 Tønsberg, Norway; 3 Norwegian National Advisory Unit for Prehospital Emergency Care (NAKOS), Oslo University Hospital, Oslo, Norway; 4 Department of Anaesthesiology, Division of Emergencies and Critical Care, Oslo University Hospital, Oslo, Norway; 5 Institute of Clinical Medicine, University of Oslo, Oslo, Norway; 6 Division of Emergency Medical Services, University Hospital of North Norway, N-9038 Tromsø, Norway

**Keywords:** Cardiopulmonary resuscitation, Bystander, Basic life support, BLS training, Competence, Chest compressions

## Abstract

**Background:**

Quality of bystander cardiopulmonary resuscitation (CPR) skills may influence out of hospital cardiac arrest (OHCA) outcomes. We analyzed how the level of CPR training related to indicators of good CPR quality and also the relationship between self-reported skills and actual CPR performance.

**Methods:**

Two hundred thirty-seven persons trained in standardized BLS curricula were divided into three groups according to the level of training: *group I* (40 h basic first aid training), *group II*, and g*roup III* (96 h advanced first aid, group III had also some limited additional life support training courses). We recorded the participants’ real-life CPR experience and self-reported CPR skills, and then assessed selected CPR quality indicators on a manikin. The data were analyzed with multivariate logistic regression. Differences between groups were analyzed with ANOVA/MANOVA.

**Results:**

Out of 237 participants, 125 had basic training (group I), 84 reported advanced training (group II), and 28 advanced training plus additional courses (group III*)*. Group II and III had shorter start-up time, better compression depth and hand positioning, higher fraction of effective rescue ventilations, shorter hands-off time, and thus a higher chest compression fraction*.* Chest compression rate did not differ between groups. The participants in group I assessed their own skills and preparedness significantly lower than groups II and III both before and after the test. In addition, group III reported higher confidence in examining the critically ill patient and preparedness in doing CPR before the manikin test than both groups I and II. However, the observed differences between groups II and III in self-reported skills and preparedness were not statistically significant after the test.

**Conclusion:**

As expected, higher levels of BLS training correlated with better CPR quality. However, this study showed that ventilations and hands-on time were the components of CPR that were most affected by the level of training. Self-assessments of CPR ability correlated well to actual test performance and may have a role in probing CPR skills in students. The results may be important for BLS instructors and program developers.

## Background

How to further improve survival after out of hospital cardiac arrest (OHCA) remains a challenge. One of the most important strategies is to identify the arrest immediately and initiate cardiopulmonary resuscitation (CPR). Large registry studies from Sweden and Denmark have recently shown how early CPR markedly improves not only good functional survival, but also reduce the amount of nursing home admissions in resuscitated patients [1–3]. Other recent studies have demonstrated that bystander CPR rates may be increased by nationwide campaigns and concomitantly lead to at least doubled survival from OHCA [1, 3–5]. Importantly, CPR quality affects OHCA outcome. Both adequate compression depth and rate as well as a high compression fraction (the proportion of the CPR time spent on chest compressions) have been shown to correlate with return of spontaneous circulation [6–8].

Consequently, CPR training for the public is of utmost importance. Bystanders trained in CPR are three times as likely to perform CPR than those untrained [9] Several different courses and options for teaching CPR are available today. Shorter CPR courses and self-instruction videos have been developed to increase the dissemination of CPR training to the public. It has been shown that chest compression quality and defibrillator use is maintained with shorter courses [10, 11]. The current guidelines therefore support simpler courses as acceptable alternatives for low-risk bystanders and in resource-limited settings [12]. However, the effect of reducing the length of CPR courses on real-life CPR quality or patient outcomes is not well known. A recent study from the Swedish OHCA registry showed increased 30 days survival when medically educated bystanders provided CPR compared to laymen bystanders, thereby suggesting that improved CPR training programmes for laypeople could improve CPR outcomes [13].

Knowledge about which parts of the training that could be improved may enable program developers to tailor courses to the needs of laypeople. However, to our knowledge, there are no controlled studies that have investigated the relationship between training level and actual performance in the different constituents of the CPR protocol. For this reason, we decided to systematically study the relationship between performance in the different parts of CPR and the length of training programs. Unfortunately, it is difficult to measure this effect directly, and thus investigators often choose to use subjective self-assessment of acquired skills as a surrogate measure. However, very little is known also about how self-assessment of skills compares with actual CPR performance.

In the present study, we aimed to analyze how the different constituents of CPR are affected by the level of training by investigating CPR providers with different length of training in otherwise comparable programs. Since we used self-assessments of skills as part of this investigation, we also aimed to systematically establish how well the participants’ self-assessment of their own skills actually related to test performance.

## Methods

### Participants

To recruit study participants with different CPR training length, but with otherwise comparable training programs, we invited 237 soldiers (both conscripted and professional) from the Norwegian armed forces. All participants were included from one single military camp, and all had received basic life support (BLS) training on an either basic or advanced level from the same courses.

The CPR training was in accordance with Norwegian guidelines based on 2010 ERC guidelines [14]. The participants were divided into three groups according to the amount of training. Group I had received a 40 h first aid course compulsory for all military personnel. The course included 5 h of CPR training, consisting of a 2 h lecture and 3 h practical training with a manikin. Participants with advanced training had received a 96 h course for future army medics, but some of these had experienced substantial additional training. For this reason, they were divided into group II (advanced course alone) and group III (advanced course and, in addition, other civilian and/or military life support courses). The advanced training curriculum included 18 h CPR training, with 4 h lectures and 14 h manikin training. Soldiers in groups II and III were also given a brief introduction to basic airway adjuncts (the oropharyngeal and nasopharyngeal tube) and assessment of respiratory distress, including needle decompression of a tension pneumothorax.

### Data collection and definitions

All participants filled out a comprehensive questionnaire designed to explore demographic parameters, additional training, and experience with CPR in real life and selected aspects of theoretical knowledge of life support. After the questionnaire, we tested the actual performance of all participants in an OHCA single-rescuer BLS manikin scenario, using the Laerdal Resusci Anne Basic (Laerdal Medical AS, Stavanger, Norway). The test included five loops of 30 compressions and two mouth-to-mouth ventilations, and the details of the scenario were unknown to the participants before they entered the room finding an unresponsive manikin on the floor. Three of the authors (ILK, MM, and JM) individually assessed the participant’s performance by direct observation, two observers squatting by the side of the manikin and one standing. The investigators did not provide any feedback during the scenario and were unaware of the participant’s level of training during the assessment. Each participant reported their self-perceived preparedness to do CPR and CPR skills on a ten-point scale using four statements about their CPR skills both before and after the manikin test.

Hands-off time and compression rate were recorded using manual counting and stopwatches. The inter-observer difference was ≤ 2 s, and we recorded the average when the assessments differed. We calculated chest compression rate from the average time used for 30 compressions during all cycles. Hand placement and compression depth were assessed and recorded independently by the observers through direct observation. Correct hand placement was defined as the lower third of the sternum in the center of the chest in all five algorithm loops, and the desired compression depth was defined as 5–6 cm vertical hand movement, measured from a point on the upper hand in centimeters during all five loops. For this reason, a vertical 20-cm scale was placed in front of the manikin for comparison, in a position where it did not provide any help to the participants.

We defined a visible rise of the manikin’s chest as adequate ventilation and defined the time used for airway management and ventilation as the time from the last compression in the loop until the start of the first compression in the following loop. Chest compression fraction was the time used on chest compressions, divided by the time from the first compression to the last ventilation in the scenario. Start-up time was the time from the participant recognized that the manikin was unresponsive and not breathing to the first performed chest compression.

### Feasibility testing of the assessment protocol

The questionnaire and the feasibility of the simple observational protocol was evaluated in a pilot study on ten nursing and medical students before the main study was initiated (data not presented). We concluded that three trained observers were needed to assess and record the scenario data and that it was feasible to measure compression depth within 1 cm inter-observer variation and time measurements within 2 s inter-observer variation. However, the method did not allow us to reliably assess ventilation volume, and insufficient or excessive ventilation volumes were not recorded. After minor corrections, we evaluated the revised questionnaire and scenario as feasible.

### Statistical analysis

We estimated the necessary sample size to 101 participants in each group, which at a two-sided 5% significance level would provide at least 90% power to detect a relevant difference in CPR skill performance.

The feasibility study had revealed a 20% difference in compression fraction and 40% in effective ventilations between groups, and we based the sample size calculations on the difference in compression fraction. A post hoc power calculation for groups II and III showed an acceptable power of 93%, given a 5% significance level and effect size of *f* = 0.25.

We calculated means and standard deviations (SD) for continuous variables, frequencies, and proportions for categorical variables, chi-square test of independence for dichotomous variables, and one-way analysis of variance (ANOVA) and multivariate analysis of variance (MANOVA) for normally distributed data. We report descriptive characteristics as means ± SDs for continuous variables and as proportions for binary variables.

We fitted multivariable logistic regression models and tested them on each outcome variable: effective ventilations, hands-off time < 7 s per loop, compression fraction > 0.7, correct hand placement, and compression depth. Model calibration was tested with the Hosmer–Lemeshow test. The exposition variable in this model was level of training (I–III) in the first model and self-reported skill rating (1–10) in the second. These models intended to show the impact of higher level of training on CPR performance in the first and association between perceived and actual CPR skills in the latter.

We adjusted for potential confounders. Time since last training has a known effect on CPR performance, and we investigated if the significant effect of the exposition variable (level of training) changed when adjusted for this variable alone, before adjusting for all variables in the model. Odds ratios (OR) with 95% confidence intervals (CI) were calculated for each outcome variable.

One-way multivariate ANOVA was conducted with three levels of training (groups I–III) as the independent variable and self-reported skill rating on a 10-point scale as the dependent variable, to test for differences between training levels and self-reported skill scores. Significant differences were analyzed with Tukey test for post hoc comparisons.

## Results

Of the included 237 soldiers, 125, 84, and 28 were in groups I, II, and III, respectively. The groups differed in several baseline characteristics (Table 1), as groups II and III had more female participants than group I. In addition, only members of groups II and III had provided CPR in real cardiac arrest situations, and groups II and III were significantly more positive to receive additional training than group I. BLS training within the last 3 months was also more common in groups II and III, but participants in all groups expressed the same willingness to perform CPR if needed in a real-life emergency.

**Table 1:** Characteristics of the participants by level of training group

	Group I	Group II	Group III	*p* value
	(*n* = 128)	(*n* = 84)	(*n* = 28)	
Age (years)	19.9 ± 1.0	19.7 ± 1.2	23.5 ± 3.0	< 0.001
Male gender	116 (90)	57 (66)	18 (64)	< 0.001
Provided CPR in real life	0	6 (7)	9 (32)	< 0.001
Time since training (months)	5.7 ± 3.0	1.9 ± 2.3	2.8 ± 2.0	< 0.001
Conscripted soldiers	128 (100)	79 (73)	0	< 0.001
Would like more CPR training	50 (39)	57 (70)	21 (75)	< 0.001
Real life lifesaving first aid	6 (4)	57 (84)	21 (75)	< 0.001
< 3 months since last training	42 (33)	71 (85)	16 (57)	< 0.001
Willing to do CPR in a real life situation	127 (99)	82 (98)	28 (100)	0.998

Group I: basic level of life support training, group II: advanced level of training, and group III: advanced level plus additional courses. Age and time since training is givens as mean ± SD, percent of *n* in parentheses for all other variables

When assessing time usage and CPR quality during the test scenario, we found a shorter time to initiation of CPR, higher fraction of effective rescue ventilations, shorter hands-off time, and thus a higher chest compression fraction in groups II and III vs group I (Table 2). In addition, compression depth and hand positioning were better in groups II and III, but chest compression rate was close to 100 compressions per minute for all groups.

**Table 2:** CPR quality indicators between groups with different training levels

	Group I	Group II	Group III	*p* value
	(*n* = 128)	(*n* = 82)	(*n* = 28)	
Compression rate^a^ First loop CPR	109 ± 29.1	107 ± 20.3	95 ± 27.9	0.036
Compression rate^a^ Second loop CPR	104 ± 26.9	106 ± 16.0	98 ± 24.5	0.365
Compression rate^a^ Third loop CPR	105 ± 26.0	106 ± 16.9	101 ± 18.3	0.603
Compression rate^a^ Fourth loop CPR	106 ± 26.3	107 ± 18.3	100 ± 21.3	0.310
Compression rate^a^ Fifth loop CPR	97 ± 26.8	113 ± 22.5	110 ± 27.3	*p* < 0.001
Effective ventilations (%)^b^	16.4	56.1	85.7	*p* < 0.001
Compression fraction^c^	0.65 ± 0.3	0.69 ± 0.3	0.74 ± 0.1	*p* < 0.001
Correct hand placement on chest (%)^d^	66.4	81.7	89.4	*p* = 0.019
Correct compression depth (%)^e^	40.6	71.4	82.1	*p* < 0.001
Total hands-off time (s)	47.0 ± 15.0	37.0 ± 12.8	30.3 ± 9.2	*p* < 0.001
Time from verified cardiac arrest to start CPR (s)	16.0 ± 12.8	3.0 ± 2.7	2.7 ± 1.2	*p* < 0.001

All results are shown as mean ± SD or %, and *p* is calculated with one-way ANOVA for continuous data and chi-square test of independence for associations between categorical data

*CPR* cardiopulmonary resuscitation

^a^Chest compressions per minute

^b^Elevation of thorax on manikin during rescue breaths

^c^Time fraction used on compressions during five loops of 30:2

^d^Lower third of sternum in centre of thorax

^e^Compression depth 5–6 cm

Because of the differences in baseline characteristics, we adjusted the results for age, gender, additional training, and time since last training, and the OR for effective ventilations, time use for ventilations, and chest compression fractions were still significantly better with advanced level of training following adjustment (Table 3). Hand placement and chest compression depth were also significantly better in groups II and III vs group I after adjustments. The most pronounced difference between the two groups (Table 1) was time since last CPR training, but we found the same significant differences when adjusting for this confounder alone, before adjustment for all variables (Table 3).

**Table 3:** Relationship between self-reported skills and observed skills

	Statements reported before manikin scenario		Statements reported after manikin scenario	
	“I feel confident to examine a critical ill patient”		“I did a good and systematic assessment of the patient”	
	OR (CI) unadjusted	OR (CI) adjusted	OR (CI) unadjusted	OR (CI) adjusted
Opens airway	1.50 (1.30–1.80)	1.36 (1.13–1.63)	1.30 (1.20–1.50)	1.10 (1.09–1.19)
Bends to check breathing	0.93 (0.70–1.30)	0.85 (0.60–1.18)	0.90 (0.70–1.20)	0.84 (0.61–1.16)
	“I am well trained in the CPR algorithm”		“I performed good quality CPR”	
	OR (CI) unadjusted	OR (CI) adjusted	OR (CI) unadjusted	OR (CI) adjusted
Effective ventilations	1.58 (1.34–1.87)	1.56 (1.29–1.89)	1.44 (1.25–1.67)	1.44 (1.20–1.70)
Compression fraction > 0.7	1.18 (1.03–1.36)	1.14 (1.02–1.33)	1.25 (1.11–1.42)	1.20 (1.05–1.37)
Time spent on two ventilations < 6 s	1.11 (0.96–1.30)	1.09 (1.11–1.18)	1.02 (0.89–1.16)	1.14 (1.05–1.20)
Compression rate 100–120 through five loops	1.21 (1.05–1.39)	1.23 (1.06–1.42)	1.03 (0.92–1.15)	1.18 (1.15–1.23)
Compression depth 5–6 cm	1.23 (1.05–1.30)	1.25 (1.06–1.47)	1.17 (1.04–1.33)	1.19 (1.04–1.37)
Correct hand placement	1.28 (1.10–1.50)	1.28 (1.08–1.50)	1.19 (1.04–1.37)	1.18 (1.02–1.37)

Skills are self-reported on a 10-point scale. The relationship between self-reported skills and actual performance is given as odds ratio (OR) and presented unadjusted and adjusted for the variables “level of training,” “time since training,” and “gender.”

*CI* 95% confidence interval, *CPR* cardiopulmonary resuscitation

Study objects on all levels of training showed overall good insight in own skills and limitations with significant associations between self-reported and actual competence for all quality indicators except “bends to check breathing”. Even the association between “I am well prepared in the CPR algorithm” and “Time spent on two ventilations” and the association between “I performed good quality CPR” and the quality indicators “Compression rate through five loops” and “Time spent on two ventilations”, that were not statistically significant before adjustment, became significant after adjustment for confounders (Table 4).

**Table 4:** MANOVA on differences between groups in self-reported skills

Self-reported skills	MANOVA (mean ± standard deviation)				Follow-up univariate ANOVA	Tukey’s post hoc test		
	Group I^a^	Group II^b^	Group III^c^	*p* value	*p* value	Group I vs. group II (*p* value)	Group III vs. group I (*p* value)	Group III vs. group II (*p* value)
“Confident to examine a critical ill patient”	4.29 ± 1.67	5.92 ± 1.92	7,00 ± 2,26	< 0.005	< 0.005	< 0.005	< 0.005	0.022
“Well trained in CPR algorithm”	6,81 ± 1.83	7.87 ± 1.72	8.75 ± 1,20	< 0.005	< 0.005	< 0.005	< 0.005	0.062
“Well prepared to do a CPR”	6,84 ± 2,04	7,60 ± 1.76	8.64 ± 1.28	< 0.005	< 0.005	< 0.005	< 0.005	0,033
“Did a systematic assessment”	3.91 ± 1.40	7.5 ± 2.12	6.1 ± 1.30	< 0.005	< 0.005	< 0.005	< 0.005	0.069
“Performed good quality CPR”	5.61 ± 1.40	8.12 ± 1.71	7.13 ± 2.10	< 0.005	< 0.005	< 0.005	< 0.005	0.091
“Would perform the same on a patient”	6.72 ± 1.50	8.73 ± 1.81	6.12 ± 2.10	< 0.005	< 0.005	< 0.005	< 0.005	0.073

Figures presented as mean ± SD

^a^Group I: basic level of life support training

^b^Group II: advanced level of training

^c^Group III: advanced level plus additional courses.

*MANOVA* multivariate analysis of variance, *CPR* cardiopulmonary resuscitation

The participants in group I assessed their own skills and preparedness significantly lower than groups II and III in all statements, both before and after test. In addition, group III reported higher confidence in examining the critically ill patient and for preparedness in doing CPR before the manikin test than both groups I and II*.* However, the observed differences in self-reported skills and preparedness between groups II and III after finishing the manikin test were not statistically significant (Table 4).

## Discussion

It is not surprising that a higher level of training leads to better CPR performance in a test scenario. However, the results presented here show that adequate airway management and ventilation skills, as well as chest compression fraction, depend significantly more on training level than other parts of CPR. Chest compression rates were similar, but also chest compression depth and hand placement were better in participants with high level of training. Importantly, all participants had received a rather brief (2–4 h) theoretical introduction to CPR, but the duration of the practical training differed significantly between basic and advanced levels of training.

Thus, we suggest that the results presented herein support the notion that enough time for practical training in these particular skills should be provided. In addition, the two groups with the most advanced course had received more training in handling respiratory distress and simple airway adjuncts. This both increased the time used on airway and ventilation training and probably gave them a broader knowledge of this topic. It is therefore likely that it may have contributed to the airway and ventilation support skills they demonstrated in the test scenario in the present study.

It may be difficult to compare groups with different levels of training because of the diversity in BLS courses. We selected Norwegian military personnel as study objects, because the armed forces train large groups of personnel in life support simultaneously. They use highly standardized curricula and training methods, and the training reaches clearly pre-defined standards at different levels. We believe that we have compared groups that are more homogenous than they would have been with other potential study objects.

Increased emphasis has been placed on shorter courses in the last years, in particular to meet the needs for BLS training in school curriculums, driver training, and at workplaces [12]. The results of the present study suggest that short courses with less time for practical manikin training may negatively affect important markers for good quality CPR. This must be weighed against the risk of less bystander CPR rates in the society if there are no simple and less time-consuming courses. However, an earlier study showed that bystander CPR of low quality (judged by a physician on the scene) gave the same outcome as no bystander CPR at all, and bystander CPR rated as good quality resulted in higher survival [15].

In the present study, the quality of almost all chest compression and ventilation parameters was better in the group with the highest level of training. Especially the ventilation part was inappropriate in group I. Maintaining an open airway and performing rescue ventilations is a complex part of the CPR algorithm, and our results support that more time should be devoted to this part during training. This is also important because it indirectly improves the chest compression fraction, which is known to increase return of spontaneous circulation and survival from OHCA [7, 8].

Indeed, airway handling is particularly challenging for laypersons; in addition, mouth-to-mouth ventilation is considered repulsive by some, and laypersons often fear that it might transfer contagious diseases [16–18]. For this reason, CPR protocols without ventilations have been suggested (CC only CPR) [19–22]. However, outcome studies comparing CC only CPR and conventional CPR are mostly observational, mainly before-after studies, and with low level of evidence [19]. Thus, the International Liaison Committee on Resuscitation (ILCOR) still recommends that “trained and willing” providers should provide rescue breaths for adult patients, based on concerns that CC only CPR might be insufficient where EMS response intervals are long and for all asphyxial cardiac arrests. Adequate airway opening and ventilations are therefore still important parts of CPR, but the knowledge about laypersons’ ability to provide successful ventilations is limited [19]. Also, international guidelines have maintained rescue breaths as part of the algorithm for trained providers because of a presumed benefit in children and in asphyxial cardiac arrest that is especially important in the less developed parts of the world [19]. Of great importance, a recently published multicentre randomized trial that compared conventional 30:2 CPR with uninterrupted CPR also supported this view [23]. All together, ventilation is still an important part of CPR, and rescue breaths should be taught. However, our findings might be used as support for CC only CPR for those with little training in ventilation management. Poor ventilations do not help, will probably lead to harm due to lower hands-on fractions, and might increase the risk of regurgitation and subsequent aspiration.

We have corrected our results for known confounders, but it is reasonable to believe that level of training also may correlate with personal interests in first aid, and perhaps a stronger motivation to help those in need of BLS. The effect of this confounder is difficult to evaluate. People choosing comprehensive courses and additional first aid training may be more likely to help because of their personal attitude. On the other hand, this can be hard to distinguish from the motivating effect of repeated and good quality training. Our study cannot fully resolve this complex question.

Even though experimental settings, like the present model, may allow us to investigate individual aspects of CPR performance, it remains to show how this relates to real life CPR situations. Some authors have pointed to the importance of teaching self-efficacy, i.e., non-technical first aid skills, including helping behavior in real-life medical emergencies [20, 24, 25]. Self-efficacy may be important, but studies about whether self-efficacy training actually changes helping behavior have given conflicting results. As direct observational studies of real-life performance and self-efficacy in CPR situations are difficult to arrange, and realistic manikin tests of large groups of CPR providers are demanding, the evaluation of acquisition of skills during training has commonly been based on the students’ own perception of skills. However, the reliability of self-assessment of the student’s own CPR training is largely unknown, and for this reason, we probed this relationship and its correlation to level of training in order to evaluate the responses from our questionnaire.

Previous educational, psychological, and sociological studies on higher education and post-graduate medical training suggest that self-reported skills do not necessarily reflect actual skills [26–28]. However, study design, the level of the students, and the field of study are all important for the relationship [26]. In our analysis of individual indicators for good quality CPR, only one skill did not show significant agreement with self-assessment. This skill was “bend to check breathing,” and the lack of correlation may theoretically be attributed to the scenario situation, as participants may have regarded as implicit that the manikin was in cardiac arrest. In contrast, the more technically demanding chest compressions and ventilations showed significant correlation. We conclude that our findings suggest that self-assessments may be used to map the students’ CPR knowledge, e. g., ahead of a course in order to tailor CPR programs to meet the individual learner’s needs.

The use of a simple manikin without automatic recording of performance parameters may seem to be a limitation. These were the same as the manikins used during training, and the setup is feasible even in low resource settings. Furthermore, the test setting was evaluated thoroughly in a small-scale pilot and we included only parameters that we found could be reliably evaluated in the described study model. We also acknowledge that it may depend on experienced facilitators whose individual accuracy and reproducibility should be assured in beforehand. Finally, we have considered the fact that the military life support training focuses on trauma, particularly on combat-related trauma, but the courses did teach CPR the same way as civilian courses. We therefore believe that the present results are relevant also for the civilian population.

## Conclusions

In the present manikin model, comprehensive BLS training resulted in better CPR quality, particularly regarding ventilations and hands-on time. We also found a significant relationship between self-perceived and actual skills.

## Abbreviations

AED: Automatic external defibrillator
ANOVA: Analysis of variance
BLS: Basic life support
CC only CPR: Chest compression only CPR
CI: Confidence interval
CPR: Cardiopulmonary resuscitation
ERC: European Resuscitation Council
ILCOR: International Liaison Committee on Resuscitation
MANOVA: Multivariate analysis of variance
OHCA: Out of hospital cardiac arrest
OR: Odds ratio
SD: Standard deviation
